# Examining the Complex Interaction Among Technological Innovation, Company Performance, and Occupational Safety and Health: A Mixed-Methods Study

**DOI:** 10.3390/ijerph21101368

**Published:** 2024-10-16

**Authors:** Gaia Vitrano, Guido J. L. Micheli, Francesca Marazzini, Valeria Panio, Angelo Castaldo, Alessia Marrocco, Stefano Signorini, Alessandro Marinaccio

**Affiliations:** 1 Department of Management, Economics and Industrial Engineering, Politecnico di Milano, 20133 Milan, Italy; guido.micheli@polimi.it (G.J.L.M.);; 2 Department of Juridical and Economic Studies, Sapienza Università di Roma, 00185 Roma, Italy; angelo.castaldo@uniroma1.it (A.C.);; 3 Department of Medicine, Epidemiology, Occupational & Environmental Hygiene, National Institute for the Insurance of Work-Related Injuries (INAIL), 00144 Roma, Italy

**Keywords:** technological innovation, Industry 5.0, occupational safety, occupational health, impact, changes, drivers, barriers, interventions

## Abstract

Technological innovation and Industry 5.0 are gaining increasing attention among researchers as they offer companies a significant competitive advantage. On the other hand, introducing these technologies also brings new risks for workers. The current literature reveals a lack of studies that effectively integrate occupational safety and health (OSH) within this emerging technological context and analyse the impacts of their use. This study aims to explore how companies interact with macro-level interventions that promote technological innovation and to understand their impact on different dimensions of company performance, including aspects related to OSH. Based on the existing literature, a research framework is presented that identifies the stakeholders involved, the inputs facilitating their interaction, and the cascading effects and changes. A mixed-methods approach was adopted by employing an in-depth survey with 89 companies responding and composed of both open-ended questions, to capture rich, qualitative insights, and multiple-choice questions, to gather quantifiable data. Two change levels have been identified: general changes and specific changes related to OSH. The analysis also delved into the main drivers and barriers that lead companies to engage with technological improvements and the multiple changes these interventions generate across company dimensions.

## 1. Introduction

Technological innovation has become a driving force in transforming industrial processes and organisational structures [1,2]. Significant transformations have been led by the spread of Industry 4.0, which has revolutionized production through cyber-physical systems, the Internet of Things (IoT), and smart manufacturing. Building on this foundation, Industry 5.0 introduces a more human-centred approach, enhancing the digital transformation and automation advances brought by Industry 4.0 [1,3]. This paradigm shift not only furthers technological progress but also addresses environmental and social dimensions, including worker well-being [3,4].

A healthy workforce constitutes an invaluable resource for efficient production processes, making occupational safety and health (OSH) issues of paramount importance. The International Labour Organisation (ILO) estimates that approximately 2.2 million people die globally each year due to work-related accidents and illnesses. Additionally, over 270 million workers suffer from non-fatal injuries [5], which lead to prolonged absences from work and life-changing consequences [6].

Thus, leveraging the potential of Industry 5.0 to provide secure working conditions for workers becomes crucial. Technological developments present opportunities to significantly improve OSH measures, but technology also introduces new types of risks [3,7]. As reported by Leso et al. [8], these technologies can make work tasks more flexible, safer, and socially inclusive. However, they can also create new health and safety risks with significant impacts on various aspects of a company’s operations [8]. The innovations guided by Industry 5.0 have the potential to raise worker awareness about OSH, ultimately facilitating their access to safer working conditions [1,9].

The following sub-sections include key topics related to OSH interventions (Section 1.1), the role of technological innovation in shaping OSH (Section 1.2), and finally, gaps in the existing research and the key research questions that will guide further investigation (Section 1.3).

### 1.1. OSH Interventions

The term “OSH intervention” refers to any physical artefact, process, procedure, skill set, or specialised knowledge that enhances health and safety, reduces or eliminates hazards for safety, or maintains, strengthens, or restores safety [10].

In the past, companies did not invest in OSH interventions because they were considered a burden rather than an added benefit. The interventions implemented within the company were typically adopted to improve productivity rather than OSH. Today, however, companies are increasingly recognizing that effective interventions can improve both OSH and productivity [11]. Many of the core principles needed for efficient OSH management—such as strong quality control, financial stability, and robust general management—are also essential for achieving broader organisational goals. Therefore, investing in OSH can bring benefits that go beyond worker well-being [12]. However, implementing effective OSH interventions remains challenging because companies must adapt to a complex and ever-changing environment shaped by organisational, economic, and technological factors [13]. Given this complexity, the numerous variables involved make it challenging to assess the impact of these factors on the effectiveness of interventions, making it difficult to predict their success [14].

Lund and Aarø [15] identified three main types of OSH interventions for accident and injury prevention in organisations, dividing changes into three categories: behaviour change, attitude change, and structural change.

- Behaviour change includes methods for directly changing behaviour without attempting to influence attitudes. Techniques like skill training and reward systems change behaviour.
- Attitude change relates to the process of changing attitudes by persuasion and information.
- Structural change refers to changing the physical environment and to modification and the availability of products.

Elements from all three major classes of preventative measures have been identified as sometimes utilized concurrently. Indeed, applying preventive measures from one area only may not be as successful as combining actions from other categories. Lund and Aarø’s model helps to identify lines of change and to understand the relationships between them.

According to Niskanen et al. [16], the interventions can occur at different levels, micro, meso, and macro, which adds degrees of organisational complexity and “levels of complexity”. In particular, the three levels can be defined as follows [16]:

- Micro-level analysis (individuals) concerns the effects on individuals, such as managers and employees, within the organisation.
- Meso-level analysis (organisations) refers to the impact of OSH measures that occur between the micro and macro levels, such as interactions within a company.
- Macro-level analysis (legislation) includes the impact of interactions caused by OSH legislation and implemented regulatory practices which influence and interact with the entire company.

Each level involves different organisational forces and variables influencing a company’s efficiency and ability to effect change. Niskanen et al. [16] presented a study related to the application of this multi-level approach, aiming to obtain a deeper understanding that allows for the examination of organisational processes from top-down and bottom-up. In their study, they underlined the need to consider a “bridge level” that moves from explanations of individual behaviour at the micro-level to explanations of organisational system characteristics at the meso-level and then to explanations of legislation at the macro-level to analyse practical implications [16].

This analysis is required to determine how much a macro intervention, such as legislation, policies, programmes, or great investments of various kinds, impacts a company. However, the literature lacks an adequate number of sources and studies that evaluate and analyse OSH interventions by considering the system as a whole, lacking particular attention to the actual impacts resulting from macro-level interventions.

Scholars of the academic literature have extensively examined the main features and evolution of OSH interventions, focusing on the three main stages of their development: design, implementation, and evaluation [17]. These phases are intrinsically linked conceptually and temporally [18]. However, while they are closely related, certain drivers and barriers may play a more critical role at specific stages of the intervention process [19].

In this context, several studies have investigated the influence of these factors on OSH interventions [20], showing how they can have a significant impact at the strategic and operational levels. However, the proposed theoretical models are often difficult to use during the design phase since they lack systematic and structured guidelines to identify the relevant mechanisms and contextual factors relevant to specific OSH interventions. This makes it complicated for practitioners to identify these elements, forcing them to rely mainly on their expertise and experience [19,21]. For this reason, many OSH interventions can only be effective under controlled conditions; when implemented in actual practice, especially in Small and Medium-sized Enterprises (SMEs), they may not work as expected [13].

Furthermore, although the three phases of OSH interventions are all equally important for their success, the literature shows that many models currently used for the evaluation phase have significant limitations and need further improvement [22]. It is important to underline that no model can be universally applied to all types of intervention. Each implementation occurs in a unique context, rendering a standardized approach ineffective [10]. OSH interventions must consider several and varied contextual factors, such as industry, culture, and organisational structure, which can influence outcomes in different ways. As a result, an intervention that is successful in one context may not be successful in another. Therefore, the context in which the intervention occurs is crucial to understanding how programmes change outcomes [23].

Finally, while interventions are generally planned with clear objectives and well-defined activities, a long-term assessment of outcomes is often lacking, with few or no indicators to monitor their success over time [24]. Many authors consider using indicators to assess performance at all stages of an intervention as a key tool for collecting qualitative and quantitative data during the planning, monitoring, training, and impact phases, thus contributing to the development of improved solutions [25]. In particular, intermediate indicators are essential for monitoring the intervention’s progress and for replacing, anticipating, and measuring potential outcomes [22]. It is equally important to determine what information needs to be subsequently monitored to ensure the continuous improvement and long-term success of the intervention [24].

Current research, however, does not provide a comprehensive model with the relevant and significant factors that practitioners can use in designing OSH interventions and a framework with a cause-and-effect chain structure since the studies focus on a limited number of factors or specific aspects.

### 1.2. Technological Innovation and OSH

Technological innovation is defined as the use of new technologies to make changes to products or services or the methods by which those products or services are manufactured [26]. It represents “a fundamental driver of economic growth and human progress” [27]; however, its implementation presents challenges. The introduction of new technologies requires a considerable commitment, often hindered by high risks, a shortage of skilled workers, insufficient funding, and regulatory constraints [28]. These obstacles are particularly evident in the case of SMEs, as they do not have the same opportunities as large businesses [29].

Investing in innovation is inherently more risky than other types of investment. Innovative projects, which aim to introduce new products, processes, or organisational practices, lead to greater uncertainty about the expected results. This can lead to failures, such as project abandonment before completion or significant delays, thus increasing investment costs. Furthermore, the relationship between innovation and progress is not straightforward; only a small percentage of businesses benefit from investing in innovation [30,31]. As a result, many companies prefer to maintain established strategies, especially when they continue to produce satisfactory results.

In recent years, Industry 4.0 has represented a further advancement in technological innovation. This new production paradigm [7,32], based on the intensive use of advanced technologies, has a significant impact on work and workers. In this scenario of industrial transformation, the study by Zorzenon et al. (2022) [33] provides an important contribution to the analysis of the effects of adopting Industry 4.0 technologies on OSH, while also introducing the more human-centred approach central to Industry 5.0. The authors offer a detailed analysis, highlighting both the benefits and the challenges associated with the implementation of these technologies. Among the key benefits is the potential to make workplaces safer and to mitigate and prevent occupational risks [34,35]. Some specific applications include excluding humans from hazardous environments through the use of industrial robots, continuous monitoring of workplace factors such as noise, temperature, and humidity to improve safety [32], improving industrial hygiene, and controlling machine safety advancements via smart devices [33].

However, the adoption of these technologies is not without risks. Some negative effects may include an increase in psychosocial risks related to the work environment, organisational work styles, pathogenic suffering from work, and work-related harm [7,32], increased stress [32], and mental fatigue [34]. Additionally, new risks may emerge in the work environment due to the use of these technologies, such as the risk of electric shocks, risks in human–robot interaction, and cyber-attacks [34]. There may also be a reduction in the level of supervision due to the adoption of these technologies [36], as well as potential health issues like poor circulation and weakened bones and muscles resulting from reduced mobility and activity (sedentarism) [37].

In this context, the implementation of Industry 4.0 technologies must take human aspects into account as an essential part [33]. Collaboration between researchers, policymakers, and stakeholders will be fundamental in ensuring a safe and optimal transition towards a more advanced production ecosystem.

In response to these needs, with the advent of Industry 5.0, the focus shifts towards a more harmonious integration between automation and the centrality of the human being. This new paradigm aims to create environments that enhance employee engagement, safety, well-being, and productivity while also strengthening the role of human learning. Unlike Industry 4.0, which primarily emphasises technological efficiency, Industry 5.0 seeks to promote greater collaboration between individuals and technological systems [38]. Despite the progress, Industry 5.0 still faces several challenges related to the effects left by Industry 4.0. Although numerous studies highlight the links between the adoption of Industry 4.0 technology and OSH [7,32], research exploring this intersection is still insufficient [7,39]. There needs to be a comprehensive and up-to-date view of the state of the art regarding the relationship between technological innovation (Industry 4.0 and 5.0) and OSH in companies [32]. This approach will be crucial in addressing emerging challenges and evaluating the effectiveness and flexibility of OSH management systems in light of a constantly evolving production context and emerging occupational risks [32].

### 1.3. Gaps and Research Questions

The literature review has provided a deeper understanding and greater awareness of the two main themes on which this study is based, OSH and technological innovation, as well as their inter-relationship. Despite the progress in technological innovation, a significant gap remains in the literature regarding its intersection with OSH [7,39]. Technological innovations are often evaluated solely for their productivity and efficiency, without considering the different levels of analysis necessary to understand the ripple effects they can have on companies [32,40]. Most research focuses on operational gains while neglecting the broader impacts on worker health and safety [41]. This leads to a limited understanding of how macro-level interventions can influence companies through cascade effects.

Furthermore, significant gaps have emerged, particularly regarding the lack of comprehensive evaluations of OSH interventions across all system levels (macro, meso, and micro), by studying significant factors with a cause-and-effect chain structure that practitioners can use in designing OSH interventions.

This study addresses the gaps identified by examining the multiple impacts of macro-level interventions that generate significant changes in companies and individuals. The main objective is to analyse how technological innovation influences various dimensions of company performance, including OSH. This study analyses macro-level interventions and how they have impacted various organisational aspects. By applying a mixed-methods approach based on an in-depth survey and evaluating the impact of various interventions more clearly and directly, this study examines the whole system from which a change in the organisation comes or from which other changes cascade. The results will contribute to a more comprehensive understanding of how innovation can be effectively aligned with OSH to foster long-term corporate success and sustainability [4,8].

To sum up, this study aims to answer the following questions:

- How do companies interact with macro-level interventions that promote technological innovation?
- What is the impact of various meso-level interventions, such as technological innovation and/or OSH, on different dimensions of company performance, including those of OSH?

This paper is structured as follows: Section 2 outlines the context in which this study was conducted, presents the research framework, and describes the research methodology; Section 3 presents the main findings; Section 4 discusses the results; and finally, Section 5 offers conclusions and recommendations for future development.

## 2. Materials and Methods

### 2.1. Context and Actors Involved

This study is part of a larger project conducted in partnership with the Italian National Institute for Insurance against Accidents at Work (INAIL, i.e., in Italian, “Istituto Nazionale Assicurazione Infortuni sul Lavoro”) and the MADE Competence Centre (CC), a research and promotion centre for Industry 4.0, supporting Italian companies in knowledge and awareness regarding various technological innovation issues, proper adoption of Industry 4.0 technologies, and the implementation of innovation projects.

INAIL continuously finances research projects from different disciplines to improve the well-being of workers and increase the overall effectiveness of prevention activities. This specific project has the objective of assessing the impact of OSH interventions that have originated from or been promoted through technological innovation, particularly in SMEs. The project aims to develop effective tools for causal analysis and long-term monitoring of the effects produced by these interventions. The project not only focuses on OSH, but it also includes other kinds of company performances, including OSH. It is crucial to understand why an intervention has specific performances, finding the connections, and not just cause-and-effect, that reliably explain the outcome.

### 2.2. Research Framework

A research framework was constructed from the objective of this study, finding the most effective way to assess the multiple impacts of a variety of interventions.

The framework is based on theoretical foundations. The study by Niskanen et al. [16], previously discussed, supports classifying interventions into three categories: macro-, meso-, and micro-levels. This multi-level model enables an analysis of the changes and actions implemented within the company, beginning with the macro-level interventions introduced by the CCs. Another important model considered for the development of the research framework is that of Lund and Aarø [15], seen before, which focuses on accident and injury prevention in organisations, dividing changes into three categories: behaviour change, attitude change, and structural change.

**Figure 1:**
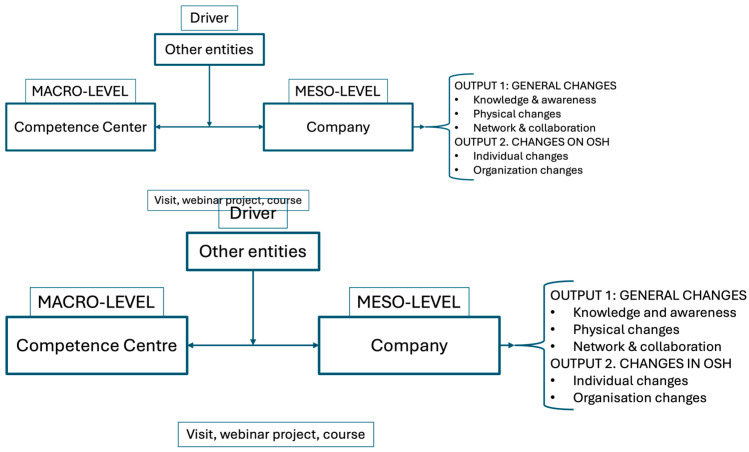
Research framework—chain of changes.

The resulting framework is represented in Figure 1. The project’s actor mapping and the process led to a number of cascading effects and changes as a result of the interaction with CCs. The straight-line arrows depict the direct interaction and effect that occurs between the CC and the company, which decides to contact the CC and utilize its offered services. Visits, webinars, projects, and courses are the primary ways in which a company can interact with the CC, taking advantage of its services. Other entities include other third-party entities, such as clients, suppliers, competitors, business associations, etc., which represent a driver for the relationship between the CC and companies. They actually have an indirect effect, influencing, enabling, and promoting the connection between the two underlying blocks.

These inputs lead to potential outputs or effects generated in cascade. They have been divided as follows:

- Output 1—generic changes in the company and its performance. These effects represent the possible generic changes that directly affect the company, its organisation, and its performance through interaction with the CC. Output 1 comprises three macro-categories: knowledge and awareness, physical change, and network and collaboration.
- Output 2—the cascade impact on OSH. These effects represent the cascade impact and changes that occur specifically in OSH due to interaction with the CC. Output 2 comprises two macro-categories: individual and organisational changes.

### 2.3. Procedure: A Mixed-Methods Approach

The methodology adopted to answer the research questions is based on a mixed-methods approach [42], integrating both qualitative and quantitative techniques, to provide a comprehensive understanding of the companies’ experiences within the CC. The study utilized an in-depth survey composed of both open-ended questions, to capture rich, qualitative insights, and multiple-choice questions, to gather quantifiable data. In particular, the open-ended questions were designed to explore the companies’ experiences related to interactions such as visits, participation in webinars, training courses, and the development of innovation processes. This approach was critical in gaining an in-depth understanding of how these experiences influenced both their overall performance and OSH outcomes. By combining qualitative and quantitative data, the mixed-methods approach ensured that the study not only captured the richness of individual company experiences but also identified broader trends and patterns that could inform future practices within the CC.

The research focused exclusively on small, medium, and large enterprises, as these profiles are more likely to engage with CCs and show interest in technological innovation. Companies usually need to be large enough, have the necessary resources, and need to know and introduce new technologies to get in touch with the CC. To obtain information about the companies, the AIDA database [43] was used, which provides detailed economic and financial information on companies operating in Italy. It contains data such as financial statements and commodity data of all active and failed Italian companies (excluding banks and insurance and public entities).

The following selection criteria were applied: companies had to belong to the manufacturing sector, have more than 10 employees, generate revenues of at least EUR 2 million, and have updated information for the years 2021, 2022, or 2023. Data extraction yielded a population of 34,422 companies, from which a sample of 1603 companies was selected to receive the questionnaire. According to the Italian Ministry of Economic Development [44], the sample was designed to reflect the Italian context, characterized by 4.8% small, 0.5% medium-sized, and 0.1% large enterprises. Micro-enterprises, which comprise the remaining 94.6%, were not included in the selection as they do not fall within the scope of interest. Therefore, the sample included 1432 small enterprises, 143 medium-sized enterprises, and 28 large enterprises.

The survey, sent by email to this sample, consisted of several questions, including two gate questions. The first asked if respondents were aware of CCs in Italy and the second gate question asked if they had contacted a CC. Based on the answers provided, three different paths were possible, which will be detailed in the next section.

After data collection, the first step in the analysis involved extracting the responses from Qualtrics, the software used to design the questionnaire, into an Excel file. During the data cleaning phase, incomplete and inconsistent responses were removed. The next phase focused on analysing the data to understand user responses, identify reasoning, and explore patterns and connections between different company groups based on their characteristics. The combined functions of Qualtrics and Excel supported the creation of graphs, facilitating the interpretation of key findings.

In-depth comparisons were made, particularly regarding the nature of the company’s interaction with CCs, categorized into four key areas of potential change: technological domain, operational infrastructure, organisational domain, and OSH. Questions were designed to capture how these interactions led to changes in these areas. To identify group similarities and differences, the analysis also considered qualitative insights from user responses, providing evidence on the lived experiences of companies and their broader effects. This cross-sectional approach aimed to capture an overview of the changes driven by the involvement of CCs, offering a broader understanding of the overall impact.

## 3. Results

**Figure 2:**
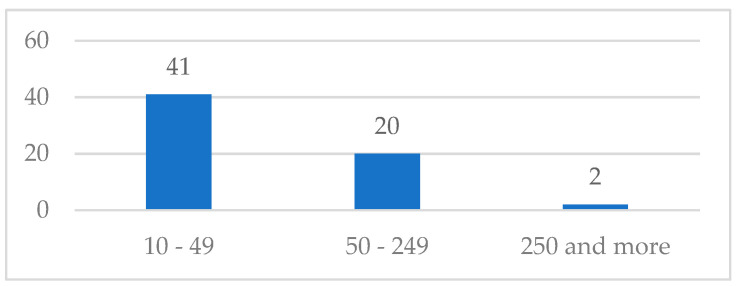
Distribution of valid survey responses by company size.

The total number of companies that responded to the survey and contributed to this study is 89. However, after cleaning the collected data, 26 questionnaires were excluded from the analysis because they were incomplete. As a result, 63 companies, including 41 small businesses, 20 medium-sized businesses, and 2 large businesses, constitute the dataset of valid responses (Figure 2). This reflects, as previously illustrated, the Italian entrepreneurial landscape, which is primarily characterized by small enterprises, with a smaller presence of medium and large enterprises.

As explained in the previous section, the user’s path is determined by two gate questions. The choices made in these questions established the path that the user followed. The answers were then divided into three categories, each based on the selected path. The three paths and their respective outcomes are described below:

- Path 1: The user selected “No” to the initial question: “Are you aware of the presence of Competence Centres on the Italian territory?” A brief description of the features and services offered by a CC was presented to these users, highlighting the importance and potential opportunities they could gain from interacting with it. The questionnaire concluded by asking whether they were interested in engaging with a CC and the reasons for their interest.
- Path 2: The user selected “No” to the question: “Did you get in touch with a CC?” The questionnaire concluded by asking for a brief explanation of the response to the previous question.
- Path 3: The user declared being aware of and having interacted with a CC, thus proceeding to answer all of the survey questions.

The results of the different paths are presented below, divided into sections corresponding to each path category.

### 3.1. Path 1

This section presents the results related to path 1, which includes users unaware of CCs. For this category, 41 responses were collected: 26 from small companies, 14 from medium-sized companies, and 1 from a large company. After providing a brief overview of the services and opportunities offered by a CC, users were asked if they were interested in collaborating. The responses were as follows:

- Twenty-two companies expressed interest in interacting with a CC;
- Twelve were not interested;
- Seven did not respond.

**Figure 3:**
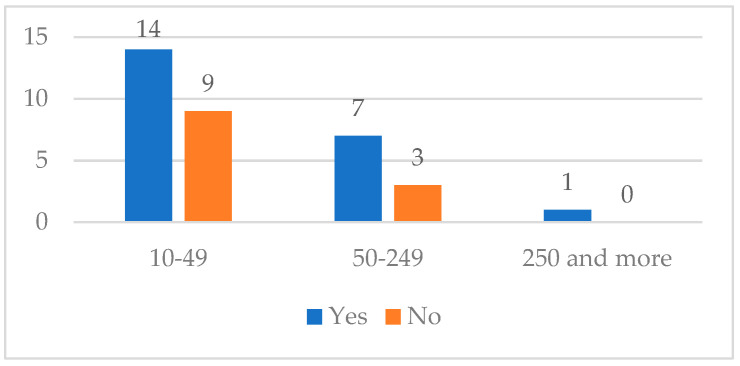
Interest in collaborating with CCs by company size.

The size of the interested and non-interested companies is illustrated in Figure 3.

The companies that responded “No” seem to have different motivations. Some provided general motivations such as “not necessary”, “there is no interest” in a deeper technological innovation, or “it is not a priority at the moment.” Other companies went into more detail, highlighting two main barriers: For some, the implementation costs of new technologies are high and not always proportionate to their size. Other companies believe they already have all of the necessary support to research, develop, and implement technological innovations.

On the other hand, the companies that responded “Yes” provided various motivations. Some highlighted the need to train specialised personnel, the opportunity for future improvements, and the development of innovation and R&D projects. The remaining companies expressed a general interest, which can be grouped into three categories: deepening their knowledge, staying updated on advancements to acquire new skills, and improving or introducing new production processes by leveraging the opportunities offered by these technologies.

### 3.2. Path 2

**Figure 4:**
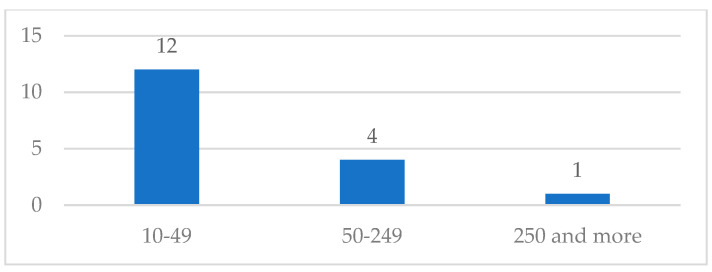
Companies aware of CCs by size.

This section presents the results of path 2, which includes users who are aware of the existence of a CC but have not yet had any contact with it. For this category, 17 responses were received. As illustrated in Figure 4, most of the responses come from small and medium-sized enterprises.

After selecting this option in the survey, users were asked to provide a brief justification for their choice, specifically the reason why they have not contacted a CC. The reasons provided were diverse. In particular, four companies mentioned that they had never really considered the topic. Meanwhile, the remaining companies reported two main reasons:

- A lack of resources: some companies stated that they did not have the time, information, resources, or sufficient personnel to dedicate to such activities.
- A lack of need: some companies did not see the need to interact with a CC, either because they already had the necessary support or knowledge, or because they preferred to invest in other areas.

### 3.3. Path 3

This section presents the results of path 3, related to companies that reported being aware of a CC and having interacted with it. This category includes five companies, all belonging to SMEs, of which three are small and two are medium-sized companies.

The first questions of the survey focus on contextualising the interaction with the CC. As shown in Table 1, these questions concern the year the companies interacted with the CC, how they became aware of it, and the reasons that prompted them to collaborate. Each of the five companies became aware of a CC through different ways, such as events, associations, regional information, the web, or word of mouth. The motivations behind collaboration also vary, although the need for renewal and improvement emerged as the main reasons.

The companies were then asked to describe their interaction with the CC, specifying the type of activities carried out and the topics discussed. As highlighted in Table 2, one company did not specify the nature of the interaction, one reported conducting a training course to facilitate the use of work support systems, and the other three mentioned innovation projects such as those focused on artificial intelligence and advanced logistics.

After contextualising and outlining the company’s experience with the CC, we asked the companies to define the general changes that have occurred since then (Table 3).

- Knowledge and awareness: all of the companies reported an increase in their knowledge and awareness through their interaction with the CC, though in different areas depending on their experiences.
- Physical changes: physical changes occurred in various areas, including the following:
  ○ Technology: new technological solutions or upgrades to existing technologies in processes, manufacturing, and assembly.
  ○ Operational infrastructure: the physical work environment and equipment/tools to support manufacturing processes.
  ○ Organisational domain: resources, information, and communication flows, company policies, procedures, processes, and production pace/efficiency.
  ○ OSH.

Table 3 shows that the changes mainly focused on the technological domain. Three companies, in particular, reported innovations related to technology, specifically focusing on the introduction of Industry 4.0 solutions (Company 1), the digitalisation of work processes through Enterprise Resource Planning (ERP) system integration (Company 2), and logistics improvements (Company 5).

- Networking and collaboration, or potential interactions with third-party entities (such as other companies, customers, suppliers, competitors, trade associations, etc.).

Table 3 shows that only one company collaborated with other entities without specifying the type of synergy that occurred.

The final questions of the survey examined the changes in OSH after the interaction with the CC. Although only Company 2, as shown in Table 3, reported an increase in knowledge and awareness regarding OSH, other companies indicated changes that occurred within their organisations in terms of OSH at later stages. It can be hypothesized that such changes are indirect consequences, arising from the acquisition of new knowledge or the implementation of physical changes, which later generated impacts in the OSH domain as well. The OSH changes are divided into OSH organisational changes and individual changes. Regarding organisational changes, as illustrated in Table 4, the main areas involved in the changes made by the companies were training, communication, and information flow, followed by prevention and evaluation of OSH performance and risks, as well as monitoring and collecting workers’ OSH data. Company 3 did not report any organisational changes, while Company 5 did not specify any modifications.

In particular, upon further investigation of the OSH changes, the users provided the following explanations:

- For Company 1: the risk evaluation was changed, updated, and improved with the introduction of new machinery.
- For Company 2: an improvement in internal communication was made due to the digitization of all processes.
- For Company 4: production flow communication procedures and training sessions have been integrated.

As for individual changes, as shown in Table 4 below, the factors most influenced by the interaction with the CC were cognitive factors. These include behaviour, attitude, resistance to change, knowledge, awareness of the importance of OSH, skills and competencies, mental stress and fatigue, motivation, and experience. Other changes related to team characteristics include composition, cohesion, coordination, and integration with non-local workers. However, Companies 1 and 3 did not observe any individual changes, while Company 5 did not provide any information on these aspects.

## 4. Discussion

The questionnaire results provide an important overview of how companies interact with macro-level interventions aimed at promoting technological innovation. In this context, the macro-level intervention in question is represented by the CC. The analysis of the responses from companies in paths one and two offers an external perspective that allows for a better understanding of the motivations behind their interaction with such an intervention. This has enabled us to identify the main barriers and constraints limiting companies’ engagement with CCs and to discuss the potential interest in contacting CCs, exploring the factors that might influence this decision.

From the analysis of the 22 positive responses from companies in path 1, it is clear that some companies desired to contact a CC to enrich their cultural background, knowledge, and awareness related to technological innovation. Other companies expressed an interest in staying updated on advancements and acquiring new skills, while others saw this as an opportunity to improve current business processes and/or introduce new ones.

On the other hand, many companies faced barriers to adopting technological innovation, primarily due to a lack of resources. It is particularly noteworthy that among the companies aware of the CC but choosing not to contact it (path two), 16 out of 17 were SMEs. As highlighted by the literature and confirmed by the questionnaire results, SMEs, being smaller and with limited resources, often lack the time, finances, personnel, and necessary information to invest in new technological projects. As noted in the questionnaire, some companies stated that the costs associated with engaging and implementing these new technologies are considerable and require investments that are not always sustainable. Moreover, the solutions offered are not always proportionate to the company’s size. This resource deficit constitutes one of the main barriers preventing companies from seizing opportunities for improvement or skill growth. As a result, macro-level interventions are not always accessible and appropriate for all types of enterprises since many lack the resources and tools to embrace these opportunities.

In addition, some companies expressed a lack of need or interest in contacting a CC. Some claim to already have all of the knowledge and skills necessary to proceed independently; others state that they have support, including external support, to solve this problem, while others prefer to focus their investments in different sectors. This lack of need or interest is likely because they already have all of the skills and resources necessary for their production, or it could be due to negligence, which prevents them from seizing the opportunity to deepen their understanding of the available and useful innovations.

To overcome these barriers, SMEs may consider collaborating with the CC. The CC is designed to promote and disseminate knowledge and to support companies in developing technological projects. It provides a wide variety of knowledge, methods, and tools in digital technologies and supports companies in addressing the digital transition towards a smart, connected, and sustainable factory. By working with the CC, SMEs could obtain the support they need to meet innovation challenges, overcome resource constraints, and fully exploit available technological opportunities.

Regarding companies engaged with CCs, the analysis shows the motivations that drove the five different companies to interact with a macro-level intervention, such as those promoted by CCs in this case. The motivations were many and varied, as were the drivers that led them to interact with the CC (regional information, trade associations, research and innovation centres, online—web and/or social media—and word of mouth). The activities carried out within it were different also according to the needs of the company that decided to investigate using CCs. Regarding the changes that companies experienced after interacting with the CC, it can be observed that in all cases, there was an increase in knowledge and awareness. However, not all companies reported physical changes. This can be explained by the fact that different inputs, such as the type of interaction and themes explored, lead to different and complex impacts, which generate further effects, giving rise to multiple potential chains of change. By analysing the causal diagrams of the different companies, it is clear that physical changes were made by companies that needed to renew parts of their infrastructure. This is the case for Companies 1, 2, and 5, which, respectively, decided to implement technological innovation through new production machinery, digitise all processes and integrate them with ERP systems for real-time progress monitoring, and make logistical improvements.

The analysis of the results also shows that only one company developed networks and collaborations as a result of the interaction with the CC. This may suggest possible aversion to sharing the information gained, a limited understanding of the benefits, or a general lack of interest in participating in collaborative interventions.

Concerning the second level of detail, it can be seen that knowledge and awareness, physical change, or both, if present, almost always directly influence the OSH domain, both at the individual and organisational level. This is evident, for example, in the case of Company 1, where, to analyse the state of Industry 4.0, the company decided to revolutionize its production machinery, enabling better risk prevention and assessment and leading to organisational changes in the OSH domain. Another key aspect is that the two macro-categories of OSH, individual change and organisational change, if both are present, influence each other reciprocally. This can be seen in Company 4, where the acquisition of new knowledge and awareness led to both organisational and individual changes in the area of OSH, improving internal communication and introducing training, as well as creating a healthy, constructive, and challenging working environment through professional development plans. These two types of OSH changes are closely related: the creation of a stimulating and motivating environment promotes healthy conditions and better integration of communication procedures between people; at the same time, training and more effective communication contribute to the creation of a healthy, safe, and constructive environment.

The analysis clearly highlights the value of examining the multiple changes generated by the implemented interventions and understanding their interconnections, which facilitates deriving insights about which inputs produce specific outputs and how these, in turn, lead to further cascading effects. These effects have an impact both on the general dimensions of the company, for example, introducing new technologies or changing the work environment, processes, and resources, and on all of the performance aspects related to them. Additionally, these changes also affect the OSH dimension, both at the organisational level, with new training sessions, a safer work environment, better monitoring, risk assessment, and better communication, and at the individual level, such as cognitive factors, teams, and all of the performance aspects related to these two OSH macro-groups.

## 5. Conclusions

This study represents a significant contribution to understanding the combination of technological innovation and OSH improvement. It is a part of a broader project funded by INAIL and in collaboration with the MADE CC.

The literature review highlighted a significant lack of studies that comprehensively integrate technological innovation and OSH topics, with a limited understanding of how technological advances affect OSH. Technological innovation is generally associated with improved productivity and business efficiency, while OSH interventions are still considered isolated activities without the potential to contribute to improving overall company performance. At the same time, there is little understanding of how a macro-level intervention can influence a firm through cascading effects, including the general impact on company performance (i.e., knowledge and awareness, physical changes, and networking and collaboration) and OSH-specific ones (i.e., individual and organisation changes).

Therefore, this study examines how firms interact with macro-level interventions that promote technological innovation and their impact on various aspects of company performance, such as technological innovation and OSH. The macro-level intervention analysed is represented by the CC. A research framework, based on theoretical foundations, was built to identify the most effective way to assess the multiple impacts of a variety of interventions. It maps the actors involved, the inputs that facilitated their interaction, and the cascading effects and changes as a result of the interaction with CCs. In particular, two levels of detail have been identified for the potential changes that may occur within the company following interaction with the CC: Output 1 (general changes) and Output 2 (changes related to OSH). Based on a mixed-methods approach, a structured survey was then created to examine the interaction with the CC and the resulting changes. From the analysis of the 89 companies participating in the survey, several conclusions emerged.

- In the analysis of companies that had not yet interacted with a CC, drivers (e.g., in-depth study, continuous updating, need to improve and innovate, training, R&D projects, networking, and collaboration) and barriers (e.g., lack of time, human resources, information, financial resources, adequate offerings, need, or interest) were identified that influence the interaction with a CC.
- In the analysis of companies with experience with a CC, it emerged that all of them reported increased knowledge and awareness. However, not all companies detected physical changes due to the variability in the inputs and topics addressed, which led to different and complex impacts, potentially generating multiple chains of changes. Regarding the second level of detail, it is noted that knowledge and awareness, physical change, or both, if present, almost always directly influence OSH, both at the individual and organisational level. Moreover, the two macro-categories of OSH, if both are present, influence each other reciprocally. The analysis highlights the importance of understanding the multiple changes generated by the implemented interventions and their interconnections, emphasising how specific inputs can lead to outputs that create cascading effects.

The analysis of the results highlights some limitations. Most of the answers come from companies that were unaware of the existence of a CC or had no direct contact with it. There are few responses from those with direct experience with a CC, which suggests the need for further research. The sample of companies that engaged with CCs is limited and not representative of all possible scenarios, which reduces the generalizability of the results. Therefore, it is essential to enlarge the sample to obtain more representative data. Although the AIDA database provides general information on companies, it does not offer specific details about their involvement with CCs. Therefore, it would be appropriate to use more targeted channels to identify companies with suitable profiles and achieve a higher response rate. Furthermore, it would be interesting and useful to delve deeper into the experiences within the CC, possibly through interviews. This would allow for a better understanding of all of the effects generated within the company as a result of contact with the CC through targeted questions and could also bring to light new and unexpected insights during the conversation. Acquiring a broader range of information on companies’ experiences and the changes implemented could help develop more in-depth analyses.

Another limitation concerns the research framework, which, although based on established elements in the literature, could benefit from comparison with experts in the field to validate its effectiveness and identify potential areas for improvement. The involvement of experts with knowledge in the implementation of macro-level interventions and the analysis of their impact could significantly contribute to refining and broadening the framework, thus improving its relevance and applicability.

## Figures and Tables

**Table 1:** Overview of interaction with CCs.

ID Company	Company Size	Year of Contact	Driver of Interaction	Motivations for Interaction
1	10–49	2022	Regional information	Analysis of technological innovation status
2	50–249	2014	Trade association	Need to improve; need to renew; training
3	50–249	2023	Association and centre for research and innovation	Networking and collaboration
4	10–49	2021	Online (web e/o social media)	Need to improve
5	10–49	2022	Word of mouth from other	Need to renew

**Table 2:** Company interaction and activities with CCs.

ID Company	Type of Interaction	Activity
1	Innovation projects	Smart monitoring and control of industrial processes; lean manufacturing; collaborative robotics and automation
2	Training course	Lean manufacturing; collaborative robotics and automation; intelligent worker assistance systems
3	Innovation projects	Artificial intelligence
4	Other	Digital twin; virtual design and product development
5	Innovation projects	Advanced logistics; product traceability

**Table 3:** General changes following interaction with CCs.

ID Company	Knowledge and Awareness	Physical Changes	Networking and Collaboration
1	Technological domain	Technology	No
2	Technological domain; OSH	Technology; operational infrastructure; organisational domain	Collaborated with other entities for other reasons
3	Operational infrastructure	No	No
4	Technological domain	No	No
5	Organisational domain	Technology	No

**Table 4:** OSH changes following interaction with CCs.

ID Company	OSH Organisation Changes	OSH Individual Changes
1	Prevention and evaluation of OSH performance and risks	None
2	Training; communication and information flow; monitoring and collection of workers’ OSH data (availability, reliability, real-time data)	Cognitive factors; team characteristics
3	None	None
4	Training; communication and information flow	Cognitive factors
5	-	-

## Data Availability

The data presented in this study are available on request from the corresponding author.
